# Supplementary material for: A UMLS-based spell checker for natural language processing in vaccine safety
Source: BMC Med Inform Decis Mak. 2007 Feb 12;7:3. doi: 10.1186/1472-6947-7-3 (PMC1805499; doi:10.1186/1472-6947-7-3)
Supplement: Additional file 13 — RAP application directory. Sets up directory for RAP (RDF application for PHP) source code [file 1472-6947-7-3-S13.gz › rap/doc/tests/Test_TraverseModel.htm]

Test Manipulate RDF Model


### RDF API for PHP V0.9.1

# Test: Traverse RDF Model

This example is part of the RAP - Rdf API for PHP documentation.

Used document: example1.rdf

`<?xml version="1.0"?>  
<rdf:RDF   
xmlns:rdf="http://www.w3.org/1999/02/22-rdf-syntax-ns#"  
xmlns:dc="http://purl.org/dc/elements/1.1/"  
xmlns:ex="http://example.org/stuff/1.0/"  
xmlns:s="http://description.org/schema/">  
<rdf:Description about="http://www.w3.org/Home/Lassila">  
<s:Creator>  
<rdf:Description rdf:nodeID="e85740">  
<rdf:type resource="http://description.org/schema/Person"/>  
<ex:Name>Ora Lassila</ex:Name>  
<ex:Email>lassila@w3.org</ex:Email>  
</rdf:Description>  
</s:Creator>  
</rdf:Description>  
</rdf:RDF>`

  
  

### 1. Parse model

`//include RDF API  
define("RDFAPI_INCLUDE_DIR", "C:/OpenSA/Apache/htdocs/rdf_api/api/");  
include(RDFAPI_INCLUDE_DIR . "RDFAPI.php");`

`// Filename of an RDF document  
$base="example1.rdf";`

`// Create a new MemModel  
$model = ModelFactory::getDefaultModel();`

`// Load and parse document  
$model->load($base);`

### 2. Get Iterator and traverse the model forward.

`// Get Iterator from model  
$it = $model->getStatementIterator();`

`// Traverse model and output statements  
while ($it->hasNext()) {  
   $statement = $it->next();  
   echo "Statement number: " . $it->getCurrentPosition()
. "<BR>";  
   echo "Subject: " . $statement->getLabelSubject()
. "<BR>";  
   echo "Predicate: " . $statement->getLabelPredicate()
. "<BR>";  
   echo "Object: " . $statement->getLabelObject()
. "<P>";  
}`

**Output:**

Statement number: 0  
Subject: http://www.w3.org/Home/Lassila  
Predicate: http://description.org/schema/Creator  
Object: e85740  
  
Statement number: 1  
Subject: e85740  
Predicate: http://www.w3.org/1999/02/22-rdf-syntax-ns#type  
Object: http://description.org/schema/Person

Statement number: 2  
Subject: e85740  
Predicate: http://example.org/stuff/1.0/Name  
Object: Ora Lassila

Statement number: 3  
Subject: e85740  
Predicate: http://example.org/stuff/1.0/Email  
Object: lassila@w3.org

### 3. And now the same backward with a jump to the middle afterwards :-)

`// Move to the last statement and print
it  
$it->moveLast();  
$statement = $it->current();`

`// Traverse model backward and print statements  
echo $statement->toString() . "<BR>";  
while($it->hasPrevious()) {  
   $statement = $it->previous();  
   echo $statement->toString() . "<BR>";  
}`

`// Jump to statement 2 and print it  
$it->moveTo(2);  
$statement = $it->current();  
echo $statement->toString() . "<BR>";`

**Output:**

Triple(bNode("e85740"), Resource("http://example.org/stuff/1.0/Email"),
Literal("lassila@w3.org"))  
Triple(bNode("e85740"), Resource("http://example.org/stuff/1.0/Name"),
Literal("Ora Lassila"))  
Triple(bNode("e85740"), Resource("http://www.w3.org/1999/02/22-rdf-syntax-ns#type"),
Resource("http://description.org/schema/Person"))  
Triple(Resource("http://www.w3.org/Home/Lassila"), Resource("http://description.org/schema/Creator"),
bNode("e85740"))  
Triple(bNode("e85740"), Resource("http://example.org/stuff/1.0/Name"),
Literal("Ora Lassila"))
